# Effects of Sowing Season on Agronomic Traits and Fatty Acid Metabolic Profiling in Three *Brassica napus* L. Cultivars

**DOI:** 10.3390/metabo9020037

**Published:** 2019-02-22

**Authors:** Xiaoyi Li, Lintao Wu, Guoliang Qiu, Tao Wang, Chunhong Liu, Yongming Yang, Bin Feng, Cun Chen, Wei Zhang, Zhibin Liu

**Affiliations:** 1 Key Laboratory of Bio-Resources and Eco-Environment of Ministry of Education, College of Life Sciences, Sichuan University, Chengdu 610065, China; 2014322040033@stu.scu.edu.cn (X.L.); BingzhenQiu@163.com (G.Q.); m15828125309@163.com (T.W.); lch1994@foxmail.com (C.L.); 2 Rape Research Institute, Guizhou Academy of Agricultural Sciences, Guiyang 550008, China; wut221@126.com (L.W.); wult12@126.com (B.F.); 3 Research Center on Flood and Drought Disaster Reduction of the Ministry of Water Resources, China Institute of Water Resources and Hydropower Research, Beijing 100038, China; starboy1986@126.com; 4 College of Chemistry and Life Science, Chengdu Normal University, Chengdu 611130, China; chencun0211@126.com; 5 College of Bioengineering, Sichuan University of Science & Engineering, Zigong 643000, China; zhangwei19840117@163.com

**Keywords:** *Brassica napus*, human consumption, fatty acid, oleic acid

## Abstract

Decreasing saturated fatty acids and increasing monounsaturated fatty acids are desirable to improve oil for food. Seed oil content and fatty acid composition are affected by genotype and environment. Therefore, we systematically analyzed the agronomic traits and fatty acid metabolic profiling of *Brassica napus* (*B. napus*) seeds at different developmental stages in high level of oleic acid (HOA), medium level of oleic acid (MOA), and low level of oleic acid (LOA) *B. napus* cultivars, both sown in winter and summer. The results showed that all winter-sown cultivars produced 20% more seed yield than the summer-sown crop. The longer growing period of winter-sown *B. napus* resulted in higher biomass production. However, the fatty acid metabolism of individual cultivars was different between winter-sown rape (WAT) and summer-sown rape (SAT). The absolute fatty acid content of LOA and MOA cultivars in WAT were significantly higher than that in SAT, but that of HOA was opposite. Importantly, the levels of monounsaturated fatty acids (18:1; 20:1) in SAT were far more than those in WAT. These data indicate the quality of oil from the HOA in SAT is more suitable for human consumption than that in WAT.

## 1. Introduction

*Brassica napus* L. (canola, rapeseed, or oilseed rape) is one of the most important oil crops worldwide. It is widely cultivated in China, India, Canada, and Europe. The yield of rapeseed in China is the highest in the world. Rapeseed provides edible oil for human consumption, protein-rich feed for animals, and raw material for industrial processes, such as biodiesel production. At least 87 million metric tons are produced yearly for human dietary and industrial uses. However, cultivated land area is decreasing, and the pace of global climate change has accelerated, leading to worldwide consumption of vegetable oil during the last decade. It ultimately has increased more than 50%, and prices have doubled.

Rapeseed yields could be seriously reduced by environmental pollution [1,2,3]. In addition to the decrease of yield, the quality of rapeseed oil is reduced by environmental stress, including drought, cold, and high salinity [4]. Increases in seed yield could be achieved if early-maturing cultivars of rapeseed can be developed for the cool climate areas or summer annual-type cultivars are grown [5,6].

Genotype and environment affect seed oil content and fatty acid profiles [7,8,9]. Air temperature has a significant effect on germination, vernalization, biomass production, growth rate, grain yield, oil quantity and quality, and oil protein [10,11,12]. Furthermore, day length is known as a major factor influencing the numbers of fertilized flowers and fully ripened seeds [13]. Daytime temperature, night temperature, and light intensity greatly affect the efficiency of photosynthesis and ultimately the biosynthesis of seed storage compounds [14]. 

The main constituents of rapeseed oils are fatty acids, including saturated (sum of palmitic, C16:0, and stearic acid, C18:0) and unsaturated (sum of oleic, C18:1, linoleic, C18:2, and linolenic acid, C18:3) acids, and triglycerides (about 3% of grain weight), which are very nutritious [15,16]. Intake of saturated fatty acids may be involved in obesity or inflammatory joint disease [17]. Erucic acid, a major derivative of the fatty acid C22:1, is also present in rapeseed oil. It has many industrial uses, including as a slip agent in plastics, or in lubricants, nylon, and cosmetics [18].

Crop productivity is challenged by decreasing cultivable land and global climate change. Suitable rapeseed cultivars are needed to meet market demands for human dietary oils and biofuel. Until now, there was very little known about the value of rapeseed in different seasons and how characteristics of climate have an effect on the fatty acid profile. In this study, we compared the differences of phenotype and fatty acids (FA) of low level of oleic acid (LOA), medium level of oleic acid (MOA), and high level of oleic acid (HOA) cultivars sown in winter (WAT) and in summer (SAT) in the two regions. We concluded that humidity and temperature parameters are ascribed to the fatty acid profile. The absolute fatty acid content of LOA and MOA cultivars in WAT were significantly higher than that in SAT. On the other hand, the levels of monounsaturated fatty acids including C18:1 and C20:1 in SAT showed far more than those in winter-sown cultivars. The LOA cultivar in WAT, with high product, is best for biofuel consumption, while HOA cultivar in SAT has the best quality of oil for human consumption.

## 2. Materials and Methods

### 2.1. Plant Material

Here, we studied three types of *B. napus* containing different levels of fatty acid (FA), termed as low oleic acid (LOA), medium oleic acid (MOA), and high oleic acid (HOA). All of them were from the “Gui You” by a semiwinter hybrid [19]. These three types of cultivars were planted in both winter (WAT) and summer (SAT) for three years. Fall-sown canola *B. napus* was grown in 2014–2017 (WAT), and summer-sown *B. napus* was grown in 2014–2016 (SAT). Field experiments were conducted at two locations affiliated with the Guizhou Rape Institute from 2014 to 2017: cultivars sown in winter in Changshun County (26.45 N. latitude, 106.99 W. longitude, the WAT site) and those sown in summer in Weining County (26.84 N. latitude, 104.31 W. longitude, the SAT site). Each location had different environmental characteristics, but both were categorized as the subtropical humid forest life zone with a monsoon climate (Table 1, Table 2 and Table 3, Supplementary Table S1). There were at least three plots at each location. When cultivars were sowed in summer, the temperature in Weining County was low enough for summer-sowing vernalization. 

All seeds of the tested plants were sown in field plots with a 0.4 m row width and 0.3 m plant spacing in each row. Each plot was 4 m in width and 5 m in length (20 m^2^). When two or three leaves had expanded, the plants were hand-thinned to obtain a uniform plant population. Normal farm management was used in cultivating the plantlets. Surface irrigation was performed twice at seeding, with a 7 to 10 d interval, to permit maximum seed germination and growth. The field was also irrigated at stemming and flowering along with N fertilization, and twice at the pod-producing and grain-filling stages. All plots in the experiment received the same amount of fertilization: during sowing, 200 kg∙ha^−1^ of ammonium biphosphate (NH_4_H_2_PO_4_), 150 kg∙ha^−1^ of urea, and 7.5 kg∙ha^−1^ borate fertilizer (12% of boron content) were banded 7.5–10 cm beneath the surface. 

Seeds in the earlier and later periods were obtained in the second and third months after pollination (MAPs) in WAT, and in the first and second MAPs in SAT, respectively. After harvest, seeds were recleaned using a 2 mm sieve, air-dried, and weighted to determine yield in the trimmed harvest area. The first flower was recorded and 75% of the plants had at least three open flowers on the main raceme.

### 2.2. Near Infrared Reflectance Spectroscopy

Near-infrared reflectance spectroscopy (NIRS) is commonly used for analysis of seed quality traits of rapeseed, such as oil, protein, glucosinolates, and fatty acid composition [20]. In this study, we used NIRS to test total protein, glucosinolates, and fatty acid for WAT and SAT. All the samples were scanned on a monochromater NIR Systems 6500 (NIR Systems, Inc., Silver Springs, FL, USA) equipped with sample autochanger. About 3–5 g intact seeds were placed in a small ring cup (*Φ* 47 cm), and reflectance spectra (log R^−1^) from 400 to 2500 nm were recorded at 2 nm intervals. 

### 2.3. Assay of FA Composition

The samples were analyzed using an Agilent 7890A-5975C gas chromatography and mass spectrometer detector and Agilent DB-WAX capillary column (30 × 0.25 mm × 0.25 µm, Agilent Co., Santa Clara, CA, USA). Helium was used as the carrier gas at a flow rate of 1 mL min^−1^. Sample (1 µL) was injected at 280 °C under a split mode (10:1). The oven temperature program was the following: 50 °C for 3 min, 10 °C min^−1^ to 220 °C, where it was held for 20 min. The ion source temperature of the mass spectrometer was 230 °C, and the temperature of the transfer line was kept at 250 °C. The electron ionization source was 70 eV. 

All fatty acids were obtained from Nu-Check Prep (Elysian, MN, USA) and were >95% pure by gas chromatography. The calibration curve as quantified with the internal standard, and the amounts of fatty acid were quantified based on the area of each compound in the total ion chromatography. There were three parallel samples in each cultivar and each sample was repeated six times. 

### 2.4. Statistical Analysis

Data are represented as the means ± SD. Statistical analysis was performed using Student’s *t* test. Values of *p* < 0.05 were considered significant.

## 3. Results

The two testing locations had different meteorological data as described (Table 1 and Table 2), including mean annual humidity, maximum and minimum temperature each day and during the reproductive period, number of days below 12 °C, and shortwave radiation. The minimum temperature in the WAT was 3 °C higher than the SAT region, while the maximum temperature in the WAT was only 1 °C lower than the SAT region (Table 1). Monthly humidity and shortwave radiation were also different (Supplementary Table S1). The WAT site was more arid than the SAT site. The maximum temperature across the lifetime of cultivars was similar, while the minimum temperature in the SAT site was 9.28 °C and decreased to 0.86 °C in the WAT site. The average shortwave radiation in the WAT site was 156.10 w·m^−2^, however, it increased twofold in the summer. 

Agronomic parameters in the same species show differences between the two sites resulting from the climate changes [21]. Table 3 shows that the bud emergences were observed at approximately 116 d in WAT for all cultivars, and at 76 d in SAT, respectively. The first flower was 138 d and 100 d after sown in WAT and SAT, respectively. The time from the first flower to the last flower lasted about 30 d in SAT, and 8 d shorter in WAT. Period of growth in WAT was about 256 d, over 8 months, while it was only 145 d for SAT. There were marked differences in flowering times and lifetimes, however, the time to seed maturity was the same, about 30 d.

**Figure 1:**
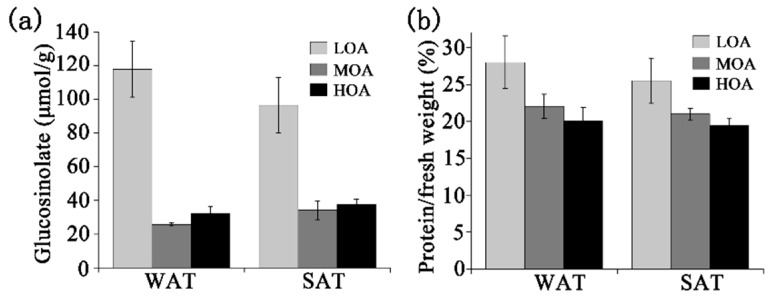
The levels of glucosinolate and protein in LOA, MOA, and HOA cultivars both in WAT and SAT through three annuals with NIRS. Values are means ± SD (n = 3 yr).

The near infrared spectroscopy (NIRS) identified glucosinolates and protein levels for three types of rapeseed in WAT and SAT. The low level of oleic acid (LOA) cultivars in WAT and SAT contained high levels of glucosinolates (Figure 1). Interestingly, the quantities of protein in three phenotype rapeseeds showed little difference for MOA and HOA cultivars, compared with LOA, indicating that there was no relationship between levels of protein and oleic acid. Together, the sowing time had an effect on the biosynthesis of glucosinolates, but not protein levels.

**Figure 2:**
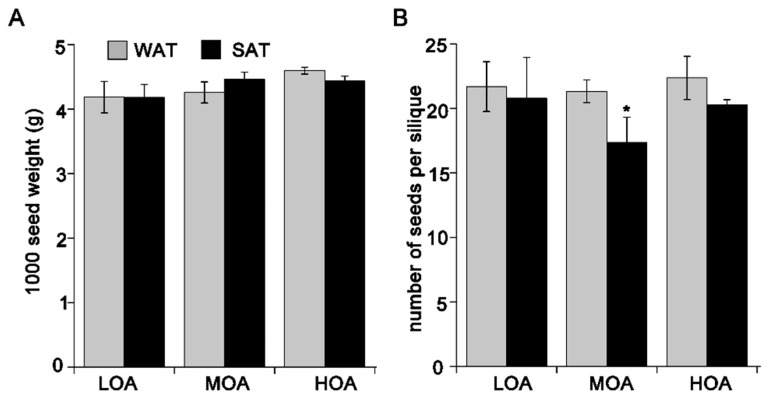
1000-seed weight (**A**) and number of seeds per silique (**B**) for all cultivars in WAT and SAT in the third annual. Values are means ± SD (n = 3 plots). * *p* < 0.05.

Next, we analyzed the effect of different sowing seasons on the agronomic parameters, including the reproductive yield and the height of cultivars. The agronomic parameters of the plants varied with cultivars in WAT and SAT (Figure 2 and Supplementary Figure S1). The seed yield was >1300 kg∙ha^−1^, in both WAT and SAT through the three test years (Table 4). The yield of rapeseed in WAT for the cultivars was higher by 100–500 kg∙ha^−1^ than that in SAT. There were significant differences between two season plantings for three cultivars. In detail, the yields of three cultivars in WAT were 1571~2039 kg∙ha^−1^, while they were 1317~1537 kg∙ha^−1^ in SAT (Table 4). However, there was no difference among cultivars in WAT or SAT. The seeds per silique for the MOA cultivars in WAT were significantly higher than those in SAT, while the HOA had no difference in the two regions. Taken together, *B. napus* planted in SAT produced fewer seeds than that in WAT.

**Figure 3:**
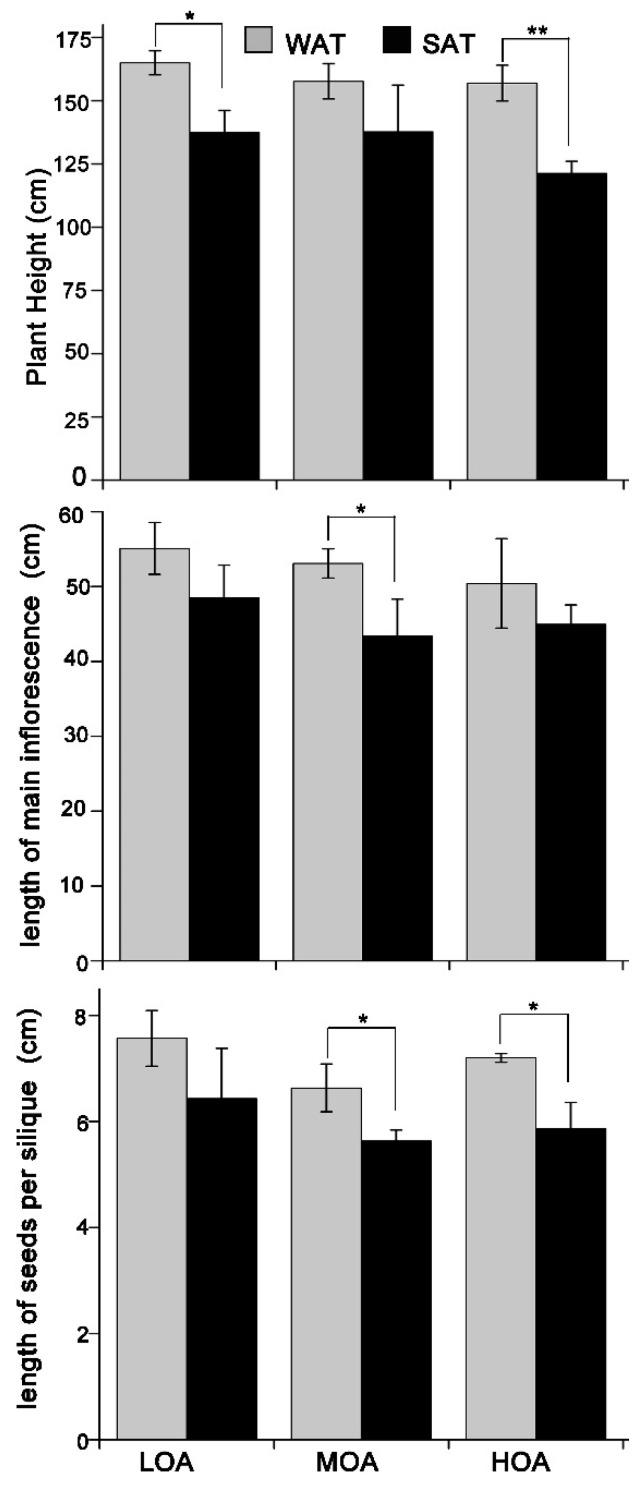
Genotype means for plant height, length of main inflorescence, and length of seeds per silique in the third year. Values are means ± SD (n = 3 plots). * *p* < 0.05, ** *p* < 0.01.

**Figure 4:**
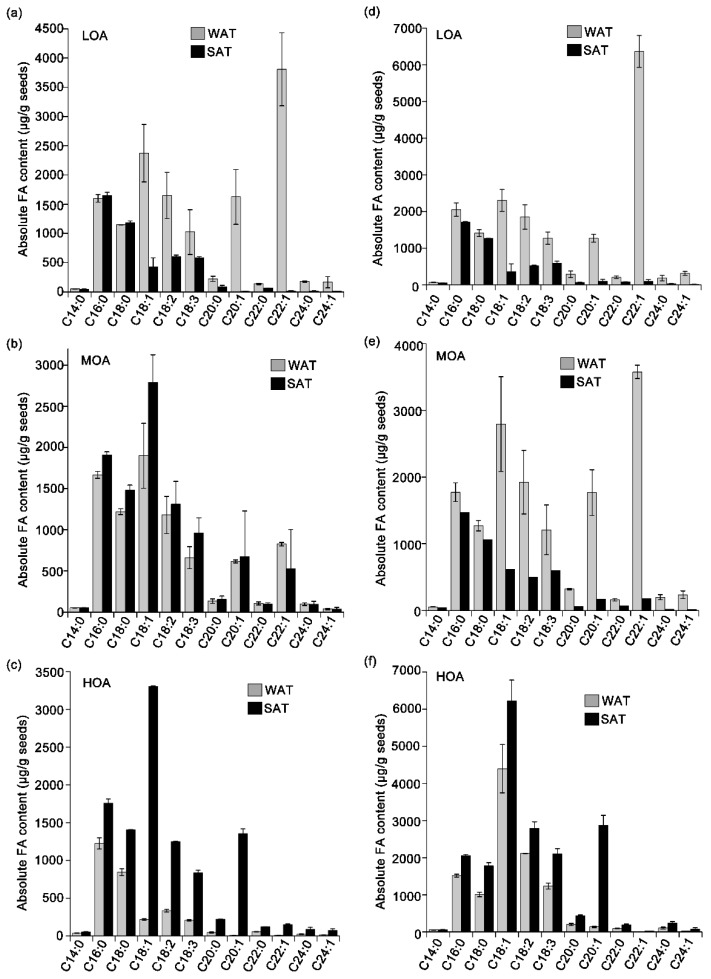
Comparisons of FA metabolic profiles in WAT and SAT among LOA, MOA, and HOA cultivars in the earlier period (**a**–**c**) and the later period (**d**–**f**). Values are means ± SD (n = 3 samples) in the third year.

In order to uncover the differences of production in WAT and SAT, the morphological diversity was observed, including height, length of main inflorescence, and length of seeds per silique (Figure 3 and Figure 4). The height of cultivars sown in summer was shorter than that of the winter-sown cultivars. Length of main inflorescence and seeds per silique in SAT moderately decreased for MOA and HOA, except for LOA. Therefore, we propose that the yield of rapeseed in WAT shows larger quantities than that in SAT, mainly contributing to the different lengths of silique in the two experimental sites. 

FAs of the rapeseeds from SAT in 2016 and WAT in 2017 were detected (Figure 4). The content of FAs are given as µg g^−1^ of seed weight for LOA, MOA, and HOA cultivars. The effects of SAT on the composition of FA species, namely myristic acid (C14:0), palmitic acid (C16:0), stearic acid (C18:0), oleic acid (C18:1), linoleic acid (C18:2), linolenic acid (C18:3), arachidic acid (C20:0), eicosenoic acid (C20:1), docosanoic acid (C22:0), erucic acid (C22:1), and tetracodanoic acid (C24:0), were investigated (Figure 4). As shown in Figure 4, cultivars with LOA sown in winter had a higher level of FAs than those sown in summer. C22:1 from the LOA cultivars in WAT had approximately 4 mg per 1 g rapeseeds in the earlier stage and increased to 4.8 mg in the later stage. For MOA cultivars, SAT planting increased the quantities of C18:1 (2800 µg g^−1^ seed), the predominant FA, in comparison to the normal planting (1800 µg g^−1^ seed). A significant increase of C22:1 in the later period was observed for the MOA cultivars, while other FAs remained at a similar level as the earlier period. The spectrum of FAs from the HOA cultivars (Figure 4e,f) was detected and showed significant differences from the LOA and MOA ones. C18:1 was the predominant FA, while linoleic C22:1 and C20:1 were the second and the third most abundant FAs, respectively. Here, C22:1 was markedly higher in SAT than in WAT, especially for the HOA cultivars (Figure 4f). The C16:0, C18:0, C18:2, and C18:3 contents in rapeseed sown in the SAT were similar in both sampling times (the earlier stage and the later stage). Notably, C14:0, C22:0, C24:0, and C24:1 were always at a low level and showed no fluctuation in both planting and the sampling time. More important, fatty acid metabolites varied as the seed matured. For example, C18:1 in MOA with summer-sown type had 2000 µg g^−1^ in the early period, and increased to 3000 µg g^−1^ in the late period of seed. An increase of C18:1 also was observed in HOA cultivars. These data demonstrate that the proportions of FAs could vary with the sowing seasons.

## 4. Discussion

*B. napus* could grow as summer-sown type, except for the winter-sown type rapeseed. In two regions, both located in Guizhou Province, three cultivars with low, medium, and high levels of fatty acid were sown in WAT or SAT. This suggests differences in microclimate, including humidity and maximum and minimum temperatures (Table 1 and Table 2). Planting rapeseed (*B. napus*) in SAT could shorten the days of growth compared to WAT (Table 3). The climate differs in these sample sites, especially the minimum temperature, which may contribute to the differences in products, agronomic characters, and fatty acid profiles.

The environment will affect global food security, including diurnal variation in temperature. Differences in glucosinolates and protein levels were not found among cultivars sown in winter and summer. However, cultivars with a low level of oleic acid contained high levels of glucosinolates (>120 m mol g^−1^), which could affect livestock and human health. Therefore, if the cultivar contains low levels of oleic acid, it cannot be used for food and some other end uses [22,23]. Glucosinolates, in combination with their hydrolyzed products, provide resistance to insects and pathogens [24,25,26]. Previous field studies showed that *B. napus* in summer conditions tends to have increased glucosinolate levels compared to the winter season [27], which our results also confirmed. However, other climatic parameters are associated with the turnover between roots and seeds, including light and relative humidity [28].

Environmental parameters influence the amount and quality of rapeseed oil, ultimately affecting public health [14]. Among the environmental parameters affecting the concentration of rapeseed oil, temperature at sowing and flowering date are among the most important. Plant height and 1000-seed weight were found to have no significant differences among cultivars both sown in winter and in summer. However, we examined the number of seeds per silique, length of the main inflorescence, and length of seeds per silique, the results indicating that there are differences among cultivars sown in winter compared to those in summer (Figure 2 and Figure 3). This result may be due to the day length and the minimum temperature influencing the numbers of fertilized flowers and fully ripened seeds [29]. In addition, the day length in summer is long, while night temperature is increased when *B. napus* flowers. Cultivars sown in winter had overall higher quantities than those sown in summer. In contrast, cultivars grown in summer had reduced seed yield. These results indicate that differences in rapeseeds are mainly due to the number of seeds per silique rather than 1000-seed weight. This may be one of the reasons that summer planting of rapeseed is not widespread. 

Climate affects agronomic characters and also coordinates the phenotype to determine the amount and quality of rapeseed oil [14,16]. Here, we analyzed the fatty acid metabolic profile among three cultivars, and all of them were winter- and summer-sown, including before and after seed maturation. The significant increase of some FA species and the significant changes altered the proportion of fatty acids in seeds (Figure 4). C18:1 increased in all cultivars planted in winter-sown type. C18:1, a monounsaturated fatty acid, is preferred in vegetable oil for biofuel production [30,31]. However, C18:2 and C18:3 had >50% increase for high level of oleic acid cultivars sown in summer, and this is consistent with unsaturated lipid substrates being required for membrane desaturation when plants are subjected to high temperatures [7,32]. In addition, linoleic C18:2 and C18:3 are not easily synthesized in the human body [33], but they are beneficial to human health due to their functions, such as anticardiovascular and anticerebrovascular functions and regulation of the human immune system [34,35]. Erucamide (C22:1), a considerably long-chain fatty acid, was found in low and medium level oleic acid cultivars sown in winter. It showed 70- and 40-fold changes, compared with those in summer, respectively. High levels of EA would be unhealthy to the human cardiovascular system [36]. Cultivars with low and medium levels of oleic acid possessed high levels of C16:0 and C18:0 both in winter and summer. Unsaturated FAs are preferred in the human diet. Therefore, we conclude that the cultivar with high levels of oleic acid should be popularized for human consumption. Like peanuts (*Arachis hypogaea* L.), the high content of OA in peanut oil is beneficial for human health and long-term storage due to its antioxidant activity [37]. However, lower levels of saturated fatty acid in the high level of oleic acid cultivar were found. Saturated fatty acids are favored for biofuel production [38]. Thus, it would be better to wildly grow cultivars containing low or medium levels of oleic acid for industrial energy.

## 5. Conclusions

Climate can affect the fatty acid metabolism of rape seeds through various mechanisms. Here, the data demonstrates that cultivars sown in winter with medium or high levels of oleic acid are suitable for use in biofuel production due to high levels of total fatty acid and high-yield genotypes. The cultivar with a high level of oleic acid in the summer-sown type is preferred for production of oil used for human consumption, contributing to its quality with high level of oleic acid, but low level of erucic acid.

## Figures and Tables

**Table 1:** The maximum temperature and minimum temperature in the SAT and WAT sites from 2014 to 2016.

Month	The SAT Site						The WAT Site					
	The Maximum Temperature (°C)			The Minimum Temperature (°C)			The Maximum Temperature (°C)			The Minimum Temperature (°C)		
	2014	2015	2016	2014	2015	2016	2014	2015	2016	2014	2015	2016
January	10.42	10.65	7.33	1.35	4.01	2.16	9.49	10.55	9.62	−0.68	1.21	−0.17
February	8.98	11.45	11.39	2.52	4.61	2.25	12.40	12.35	7.23	0.41	2.06	−0.56
March	14.28	14.69	15.33	7.12	8.07	7.95	17.88	19.38	17.35	4.72	5.64	4.67
April	20.75	21.43	21.66	13.75	11.95	13.61	20.99	18.41	20.77	10.00	9.11	8.99
May	22.13	24.19	24.04	14.38	16.42	15.73	21.70	22.46	21.88	11.42	12.81	11.89
June	24.70	24.76	27.37	18.93	19.52	19.84	21.87	22.50	23.54	14.79	14.97	14.66
July	27.26	26.56	29.36	19.88	18.87	20.99	22.61	22.48	24.52	15.37	14.63	16.62
August	27.35	25.93	28.86	19.13	19.02	20.19	22.88	21.24	24.73	15.38	14.75	15.91
September	26.61	23.16	25.94	18.27	17.35	16.85	22.51	19.70	19.42	14.70	13.69	13.55
October	22.42	21.90	22.30	13.83	13.69	15.16	19.02	18.92	19.74	10.62	10.47	11.86
November	14.46	18.58	15.61	9.72	11.89	9.16	12.71	18.85	16.91	6.08	7.08	7.01
December	10.39	9.87	12.78	3.73	4.70	5.63	7.95	9.94	12.04	0.64	2.69	3.04
Annual	19.14	19.43	20.16	11.88	12.51	12.46	17.67	18.07	18.14	8.62	9.09	8.96

**Table 2:** Humidity, temperature parameters (°C), radiation in the reproduction stage.

	Mean Annual Humidity	Max. Temp (°C)	Min. Temp (°C)	Days Below 12 (°C)	Mean Shortwave Radiation (w·m^−2^)
WAT	2.41	30.90	0.86	78	156.10
SAT	4.64	29.40	9.28	5	307.73

**Table 3:** Effect of planting date on genotypes across three years for the mean of all agronomic traits collected.

	WAT	SAT
Germination	20 October	8 May
Bud emergence (days)	116 ± 5	76 ± 3
First flower (days)	138 ± 4	100 ± 2
Last flower (days)	176 ± 5	130 ± 5
Maturity of seeds (days)	201 ± 7	154 ± 8
Days of reproduction	92 ± 5	85 ± 4
Days of growth	256 ± 10	145 ± 8

**Table 4:** Seed yield of cultivars with LOA, MOA, and HOA in WAT and SAT for three years.

Year	Seed Yield in WAT (kg∙ha^−1^)			Year	Seed Yield in SAT (kg∙ha^−1^)		
	LOA	MOA	HOA		LOA	MOA	HOA
2014.10–2015.05	1812.89 ± 62.79	1571.36 ± 18.26	1808.01 ± 25.04	2014.05–2014.10	1437.16 ± 89.91	1537.21 ± 58.86	1400.55 ± 32.95
2015.10–2016.05	1810.50 ± 56.17	1791.00 ± 64.35	1693.38 ± 30.25	2015.03–2015.10	1317.62 ± 89.9	1478.66 ± 27.78	1500.60 ± 74.11
2016.10–2017.05	1854.28 ± 10.89	1683.60 ± 10.62	2039.85 ± 22.75	2016.05–2016.10	1484.13 ± 58.19	1446.92 ± 50.43	1352.37 ± 14.03
Mean	1825.89 ± 24.62	1681.99 ± 109.83	1847.08 ± 176.51	Mean	1412.08 ± 176.51 **	1487.60 ± 45.80 *	1417.84 ± 75.61 *

* *p* < 0.05; ** *p* < 0.01.

## Supplementary Materials

The following are available online at https://www.mdpi.com/2218-1989/9/2/37/s1, Figure S1: Genotype means for all variables (Plant height, length of main inflorescence, length of seeds per silique and 1000 seed weight) in the first annual (A) and the second annual (B) in the SAT and WAT sites. Values are means ± SD (n = 3), Table S1: Meteorological parameters including precipitation (mm), shortwave radiation (W·m^−2^) in the SAT and WAT sites during the experiments.

## Abbreviations

LOA, low level of oleic acid; MOA, medium level of oleic acid; HOA, High level of oleic acid; WAT, Winter-sown rape; SAT, Summer-sown rape; GC-MS, gas chromatography mass spectrometry; NIRS, near-infrared reflectance spectroscopy.
