# Supplementary material for: Effects of Sowing Season on Agronomic Traits and Fatty Acid Metabolic Profiling in Three Brassica napus L. Cultivars
Source: Metabolites. 2019 Feb 22;9(2):37. doi: 10.3390/metabo9020037 (PMC6409595; doi:10.3390/metabo9020037)
Supplement: Supplementary file 1 [file metabolites-09-00037-s001.zip › Supplementary Figure 1.pptx]

## Slide 1
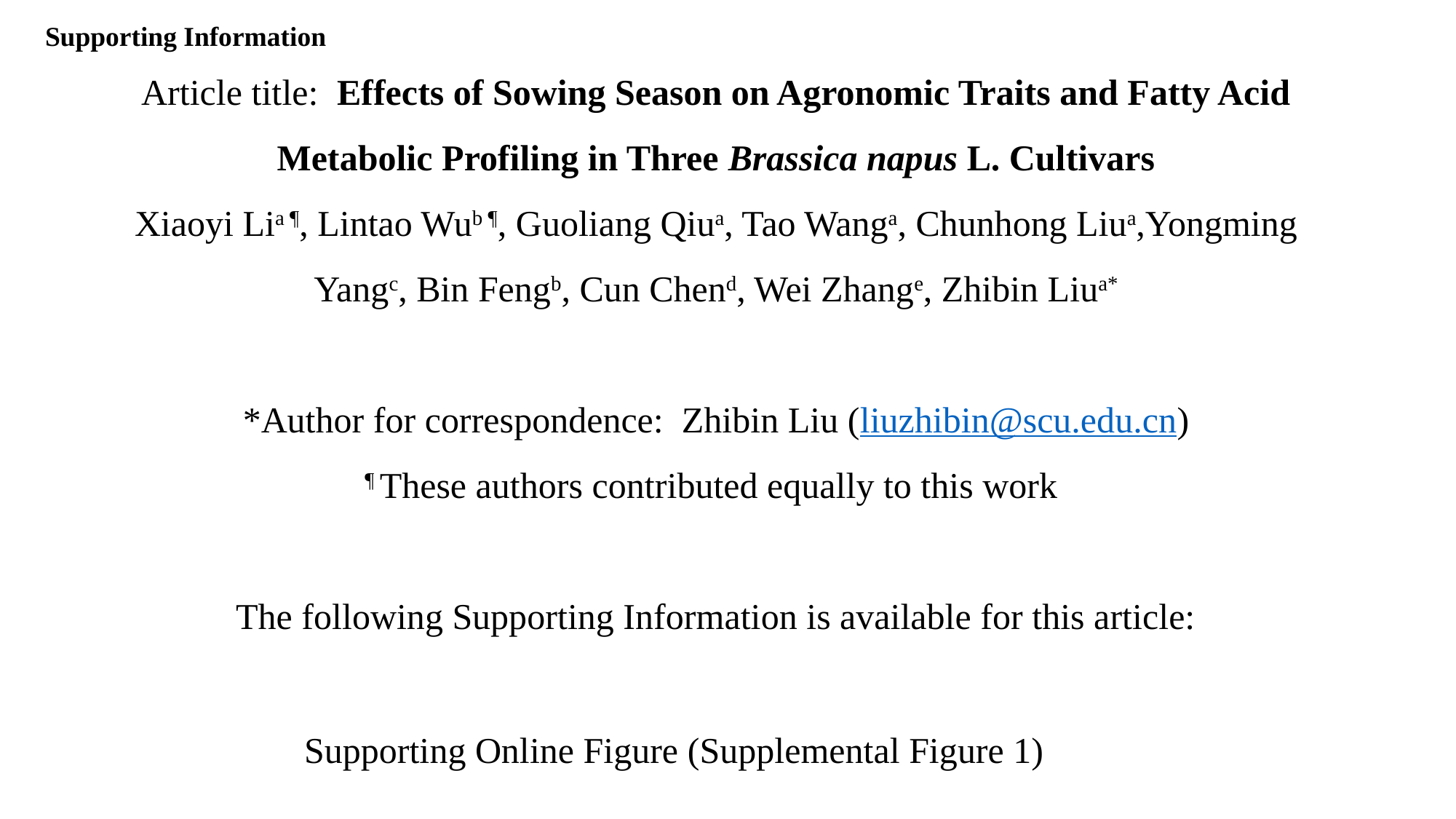

Supporting Information
# Article title: Effects of Sowing Season on Agronomic Traits and Fatty Acid Metabolic Profiling in Three Brassica napus L. Cultivars Xiaoyi Lia ¶, Lintao Wub ¶, Guoliang Qiua, Tao Wanga, Chunhong Liua,Yongming Yangc, Bin Fengb, Cun Chend, Wei Zhange, Zhibin Liua*   *Author for correspondence: Zhibin Liu (liuzhibin@scu.edu.cn) ¶ These authors contributed equally to this work The following Supporting Information is available for this article:
Supporting Online Figure (Supplemental Figure 1)

## Slide 2
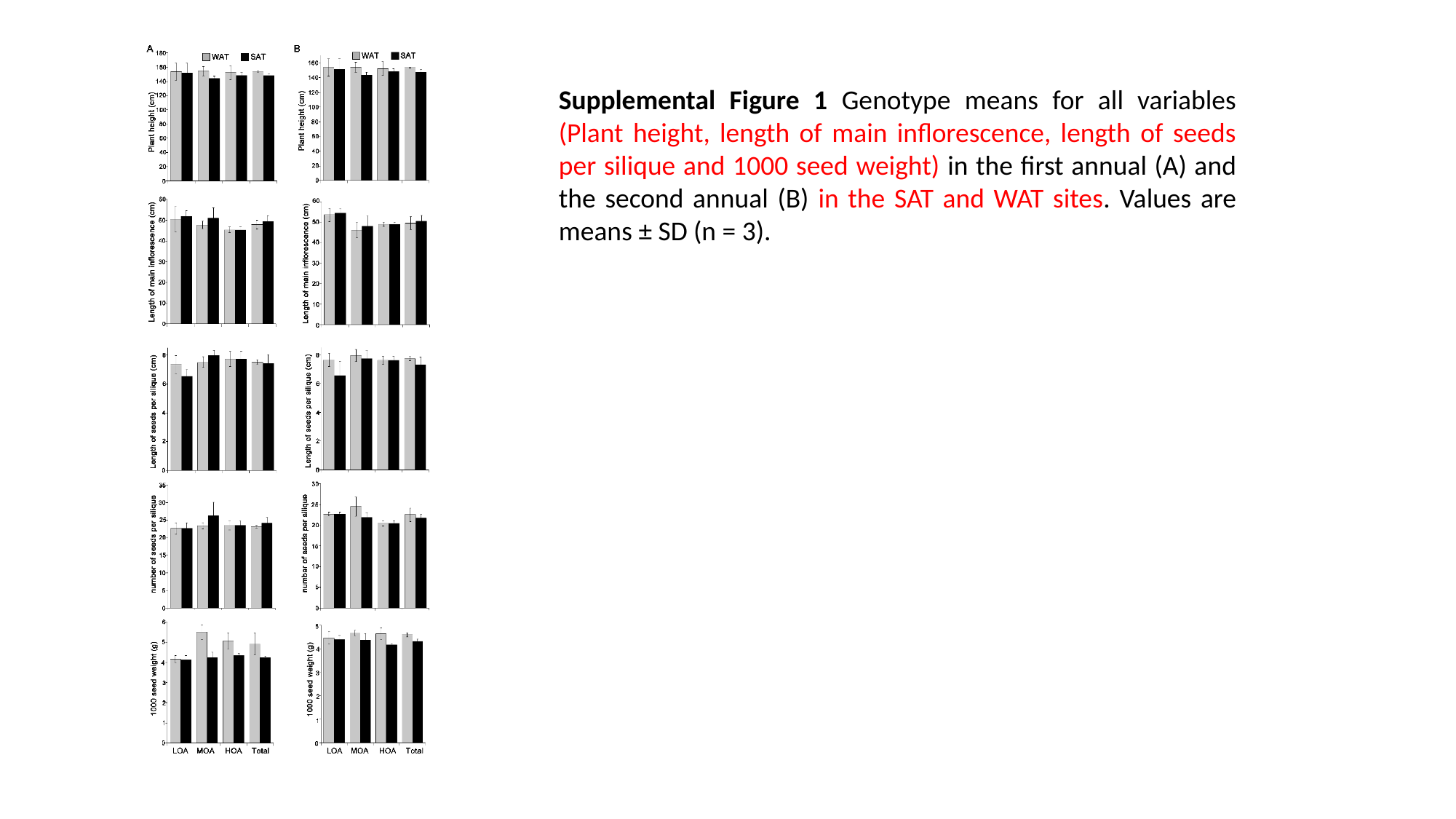

Supplemental Figure 1 Genotype means for all variables (Plant height, length of main inflorescence, length of seeds per silique and 1000 seed weight) in the first annual (A) and the second annual (B) in the SAT and WAT sites. Values are means ± SD (n = 3).
