# Supplementary material for: Effects of Sowing Season on Agronomic Traits and Fatty Acid Metabolic Profiling in Three Brassica napus L. Cultivars
Source: Metabolites. 2019 Feb 22;9(2):37. doi: 10.3390/metabo9020037 (PMC6409595; doi:10.3390/metabo9020037)
Supplement: Supplementary file 1 [file metabolites-09-00037-s001.zip › Supplementary Table 1.pdf]

Supplementary Table 1 Meteorological parameters including precipitation (mm), shortwave radiation ($W.m^{-2}$) in the SAT and WAT sites during the experiments.

Month	The SAT site						The WAT site					
	Precipitation (mm)			shortwave radiation ($W.m^{-2}$)			Precipitation (mm)			shortwave radiation ($W.m^{-2}$)		
	2014	2015	2016	2014	2015	2016	2014	2015	2016	2014	2015	2016
January	0.25	1.16	0.80	151.32	127.05	117.95	0.14	0.68	0.05	160.97	149.94	160.96
February	0.97	0.37	0.20	146.56	154.54	167.81	0.47	0.08	0.15	191.10	196.25	163.60
March	1.94	0.74	1.95	179.93	161.35	173.30	0.67	0.33	0.67	250.10	243.71	227.04
April	2.75	3.65	4.62	195.20	207.92	192.50	1.63	2.98	2.04	269.60	224.36	243.84
May	5.07	8.67	5.19	198.18	181.21	206.54	2.30	1.29	4.26	255.47	242.44	246.31
June	5.79	12.37	6.13	171.03	164.44	203.11	4.59	6.68	7.32	205.27	214.83	237.35
July	12.35	2.02	3.76	211.13	208.25	222.76	8.08	5.13	4.29	214.57	239.88	227.62
August	4.59	4.79	4.39	206.38	190.93	208.85	10.67	9.73	3.12	222.14	198.53	231.86
September	2.62	3.52	1.00	194.76	157.39	186.17	4.44	2.79	3.89	203.11	159.32	163.92
October	3.33	3.57	1.94	168.70	157.46	151.57	2.54	4.83	1.00	181.65	182.72	173.47
November	3.16	1.39	2.32	122.67	132.64	125.26	0.98	0.09	0.49	140.29	171.33	158.26
December	0.16	1.69	1.19	128.08	116.46	122.00	0.07	0.48	0.48	129.85	132.12	140.94
Annual	3.58	3.66	2.79	172.83	163.30	173.15	3.05	2.92	2.31	202.01	196.29	197.93
